# Molecular detection of *Ehrlichia* spp. in ticks parasitizing wild lagomorphs from Spain: characterization of a novel *Ehrlichia* species

**DOI:** 10.1186/s13071-022-05600-4

**Published:** 2022-12-14

**Authors:** Susana Remesar, Sabrina Castro-Scholten, Patrocinio Morrondo, Pablo Díaz, Débora Jiménez-Martín, Carlos Rouco, Leonor Camacho-Sillero, David Cano-Terriza, Ignacio García-Bocanegra

**Affiliations:** 1 Investigación en Sanidad Animal: Galicia (Grupo INVESAGA), Facultad de Veterinaria, Universidade de Santiago de Compostela, Lugo, Spain; 2 Departamento de Sanidad Animal, Grupo de Investigación en Sanidad Animal y Zoonosis (GISAZ), UIC Zoonosis y Enfermedades Emergentes ENZOEM, Universidad de Córdoba, 14004 Córdoba, Spain; 3 Departamento de Botánicía, Ecología y Fisiología Vegetal, Universidad de Córdoba, 14014 Córdoba, Spain; 4 Programa Vigilancia Epidemiológica Fauna Silvestre (PVE), Consejería de Sostenibilidad, Medio Ambiente y Economía Azul, Junta de Andalucía, Málaga, Spain; 5 Centro de Investigación Biomédica en Red de Enfermedades Infecciosas (CIBERINFEC), Instituto de Salud Carlos III (ISCIII), Madrid, Spain; 6 Department of Plant Biology and Ecology, University of Seville, Seville, Spain

**Keywords:** Anaplasmataceae, Wild rabbits, Iberian hares, Ticks, Iberian Peninsula

## Abstract

**Background:**

Several species belonging to the genus *Ehrlichia* are considered pathogenic for animals and humans. Although wildlife are known to play an important role in the epidemiology of these bacteria, information on the role of wild lagomorphs in their sylvatic cycle is limited. Thus, the objective of the present study was to assess the occurrence of *Ehrlichia* spp. in ticks collected from wild lagomorphs in Spanish Mediterranean ecosystems.

**Methods:**

A total of 1122 pooled ticks (254 pools) collected from 506 wild rabbits (*Oryctolagus cuniculus*) and 29 Iberian hares (*Lepus granatensis*) were analysed using a nested PCR assay targeting the partial *groEL* gene. *Ehrlichia* spp*.*-positive samples were further subjected to a second PCR assay targeting 16S rRNA.

**Results:**

Three (1.2%) tick pools comprising *Rhipicephalus pusillus* collected from nine wild rabbits were positive for *Ehrlichia* spp. All the *Ehrlichia* DNA sequences were identical, and use of sequence and phylogenetic analyses allowed us to identify a novel *Ehrlichia* species.

**Conclusions:**

We provide evidence that a novel *Ehrlichia* species, named herein as ‘*Candidatus* Ehrlichia andalusi’, which may be of concern for animal and public health, is circulating in *R. pusillus* in Spanish Mediterranean ecosystems. Further studies are warranted to assess the epidemiology, pathogenicity and zoonotic potential of this *Ehrlichia* species.

**Graphical Abstract:**

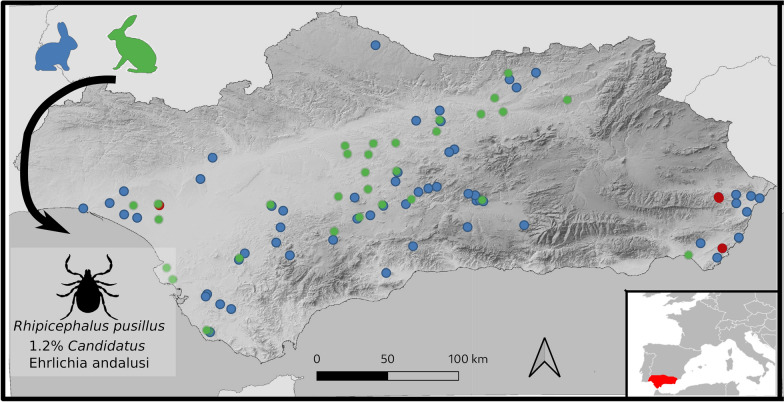

## Background

The incidence of tick-borne pathogens has increased worldwide during the last decades [1]. This emergence, or re-emergence, may be related to climate change, global travel, changes in land use (urbanization, deforestation, habitat fragmentation, etc.), or an increase in outdoor activities, among other factors [2]. Scientists and health authorities are very concerned about tick-borne agents of disease, so increased diagnostic pressure may also explain the increased detection of these pathogens in vectors, other animals and humans [3].

Among the wide variety of tick-borne pathogens, those belonging to the family Anaplasmataceae are of special interest due to their zoonotic potential and worldwide distribution [4]. Within this family, the genus *Ehrlichia* is of major concern. Several species belonging to this genus are considered pathogenic for both domestic and wild animals, such as *Ehrlichia canis, Ehrlichia chaffeensis* and *Ehrlichia ruminantium* [5]. In addition, *Ehrlichia canis, Ehrlichia chaffeensis, Ehrlichia ewingii* and *Ehrlichia muris* have been shown to be zoonotic [6].

Since transovarial transmission of *Ehrlichia* spp. has not been demonstrated in ticks [7], it has been suggested that wildlife may play an important role in the epidemiology of these pathogens [8]. Although there is an increasing number of studies providing information on the presence and prevalence of *Ehrlichia* spp. in domestic and wild ruminants and their ticks, data on the epidemiology of these bacteria in ticks collected from wild lagomorphs are still scarce. Therefore, the aim of the present study was to molecularly determine the occurrence of *Ehrlichia* spp. in pools of ticks parasitizing wild rabbits (*Oryctolagus cuniculus*) and Iberian hares (*Lepus granatensis*) in Mediterranean ecosystems in southern Spain.

## Methods

### Sample collection

Between October 2016 and August 2020, a total of 1122 ticks were collected from 506 wild rabbits (total number of rabbits examined = 1304) and 29 Iberian hares (total number of hares examined = 58). These specimens were identified in a previous study [9] as *Rhipicephalus pusillus, Rhipicephalus sanguineus* sensu lato, *Haemaphysalis hispanica, Hyalomma lusitanicum* and *Ixodes ventalloi*. The ticks were kept frozen at − 20 °C until examination.

For the detection of *Ehrlichia* spp. DNA, ticks collected from wild rabbits and Iberian hares hunted in the same hunting area were pooled according to species, development stage and host species [9]. The number of pools for each tick species is summarized in Table 1.

**Table 1:** Percentage of pools positive to *Ehrlichia* spp. and maximum likelihood estimation (MLE) from wild rabbits and hares when considering the tick development stage

	Stage of development						Total
	Nymph		Adult (female)		Adult (male)		
	%	MLE	%	MLE (95% CI)	%	MLE	%
Wild rabbits							
* Rhipicephalus pusillus*	0/65 (0%)	–	3/54 (5.6%)	1.25 (0.31–3.20)	0/50 (0%)	–	3/169 (1.78%)
* Rhipicephalus sanguineus* s.l.	–	–	–	–	0/2 (0%)	–	0/2 (0%)
* Haemaphysalis hispanica*	0/13 (0%)	–	–	–	–	–	0/13 (0%)
* Hyalomma lusitanicum*	0/23 (0%)	–	–	–	–	–	0/23 (0%)
* Ixodes ventalloi*	–	–	0/4 (0%)	–	0/2 (0%)	–	0/6 (0%)
Hares							
* R. pusillus*	0/3 (0%)	–	0/11 (0%)	–	0/10 (0%)	–	0/24 (0%)
* R. sanguineus* s.l.	–	–	–	–	0/11 (0%)	–	0/11 (0%)
* H. lusitanicum*	0/4 (0%)	–	0/2 (0%)	–	–	–	0/6 (0%)

### Molecular analyses

Tick DNA was extracted using a commercial kit (High Pure PCR Template Preparation Kit; Roche Diagnostics, Mannheim, Germany), following the manufacturer’s instructions. *Ehrlichia* spp. DNA was detected by a nested PCR assay targeting a partial fragment of the *groEL* gene [10, 11] Amplicons of the expected size were purified, sequenced, aligned and edited as previously reported [9]; consensus sequences were then scanned against the GenBank database using the Basic Local Alignment Search Tool. All *Ehrlichia* spp.-positive samples were further subjected to a second PCR protocol targeting the 16S rRNA of these bacteria [5, 12, 13]. The PCR products were processed, sequenced and analysed again, as previously described.

A phylogenetic analysis was carried out using MrBayes 3.2.7 software [14] by Bayesian approach with Markov Chain Monte Carlo sampling (10,000,000 generations sampling every 1000 steps). A Hasegawa-Kishino-Yano substitution model with gamma-distributed rate variation across sites was used for the analysis of *Ehrlichia* spp. sequences at the *groEL* and 16s rRNA genes. The model was selected based on Akaike information criterion values using the free software jModelTest v.2.1.10 [15]. The tree was visualized and edited using FigTree 1.4.3 (http://tree.bio.ed.ac.uk/software/figtree/).

### Statistical analysis

Maximum likelihood estimation was used to estimate the prevalence of *Ehrlichia* spp. in pooled *R. pusillus* [16]. Statistical analyses were performed using the statistical software R 4.2.1 [17] and the functions llprevr and dprev [16].

## Results and discussion

Only three out of the 254 (1.2%) tick pools (maximum likelihood estimate 0.3%, and 95% confidence interval 0.1–0.7) yielded positive results with respect to targeting of the *groEL* partial gene (Table 1). These results revealed that *Ehrlichia* spp*.* were not prevalent in the ticks collected from the wild lagomorphs from Mediterranean ecosystems of southern Spain, which suggests that these ticks probably do not play an important role in the sylvatic cycle of these pathogen*s.*

**Fig. 1:**
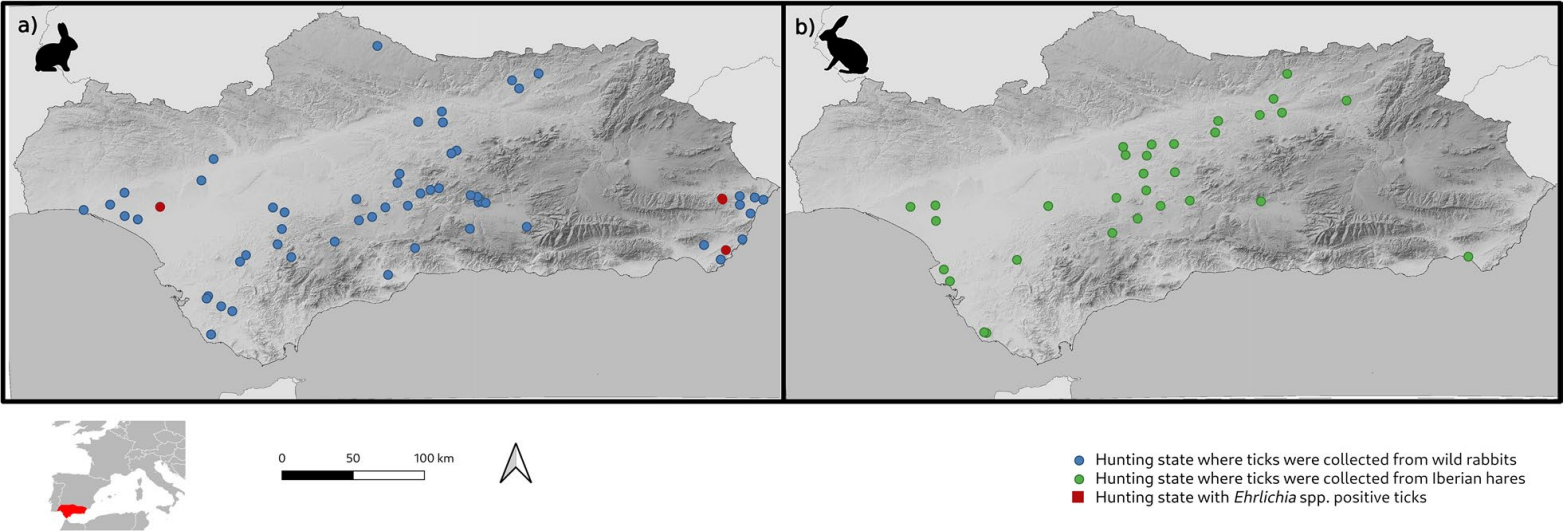
Distribution of the tick samples collected from wild rabbits (**a**) and Iberian hares (**b**)

All the positive pools comprised female *R. pusillus* obtained from nine rabbits hunted in three hunting areas in eastern and western Andalusia (Fig. 1), and represent, to the best of our knowledge, the first report of *Ehrlichia* spp. in *R. pusillus*. To the best of our knowledge, there is only one previous report of Anaplasmataceae in this tick species, where 1.8% of *R. pusillus* collected from horses in France were found to be positive for *Anaplasma phagocytophilum* [18]. *Ehrlichia* DNA was not detected in the other tick species collected from the wild rabbits or the Iberian hares. However, *R. sanguineus* sensu lato is known to be involved in the transmission of numerous pathogens, including *E. canis* [19], and questing *I. ventalloi* from Portugal and Spain were found to harbour Anaplasmataceae, including *A. marginale* and *A. phagocytophilum* [20–22]. The vectorial competence of *H. lusitanicum* in the transmission of *Ehrlichia* spp. is poorly understood; however, DNA of these bacteria was detected in *H. lusitanicum* from Italy [23]. Finally, little is known about tick-borne pathogens in *H. hispanica* [24].

The sequences identified in this study were deposited in GenBank under accession numbers OP490270 and OP502086. Sequence analysis revealed that all the *Ehrlichia* spp. isolates were identical to each other at both the *groEL* and 16S rRNA genes. For the *groEL* gene, all the sequences had a percentage nucleotide identity between 91.5% to 91.7% when compared to uncultured *Ehrlichia* sp. clone Tajikistan sequences KJ930191 and KJ930192 obtained from *Hyalomma anatolicum* from Tajikistan [25]; 91.7% identity with sequences MW054555 and MW054557 deposited for *Ehrlichia* sp. isolate YNT obtained from *Rhipicephalus annulatus* and *Rhipicephalus geigyi* from Guinea [26] was also found. In addition, the nucleotide sequences at the 16S rRNA partial gene showed a percentage identity ranging from 99.4 to 99.7% when compared to several deposited sequences of uncultured *Ehrlichia* spp*.* (AF311968, AY309970, KJ410257, KX987325, KX577724, KY046298, MH250197, MT258392 and OK481113) from different species of *Hyalomma*, *Rhipicephalus* and *Haemaphysalis* from African and Asian countries, including Angola [27], China [28, 29], Japan [30, 31], Malaysia [32], Niger [33] and Pakistan [34]. The degree of similarity between the *Ehrlichia* species at the 16S rRNA gene could indicate that it is not an appropriate gene for discriminating between species of this genus, similar to previous conclusions for other bacterial genera [35].

**Fig. 2:**
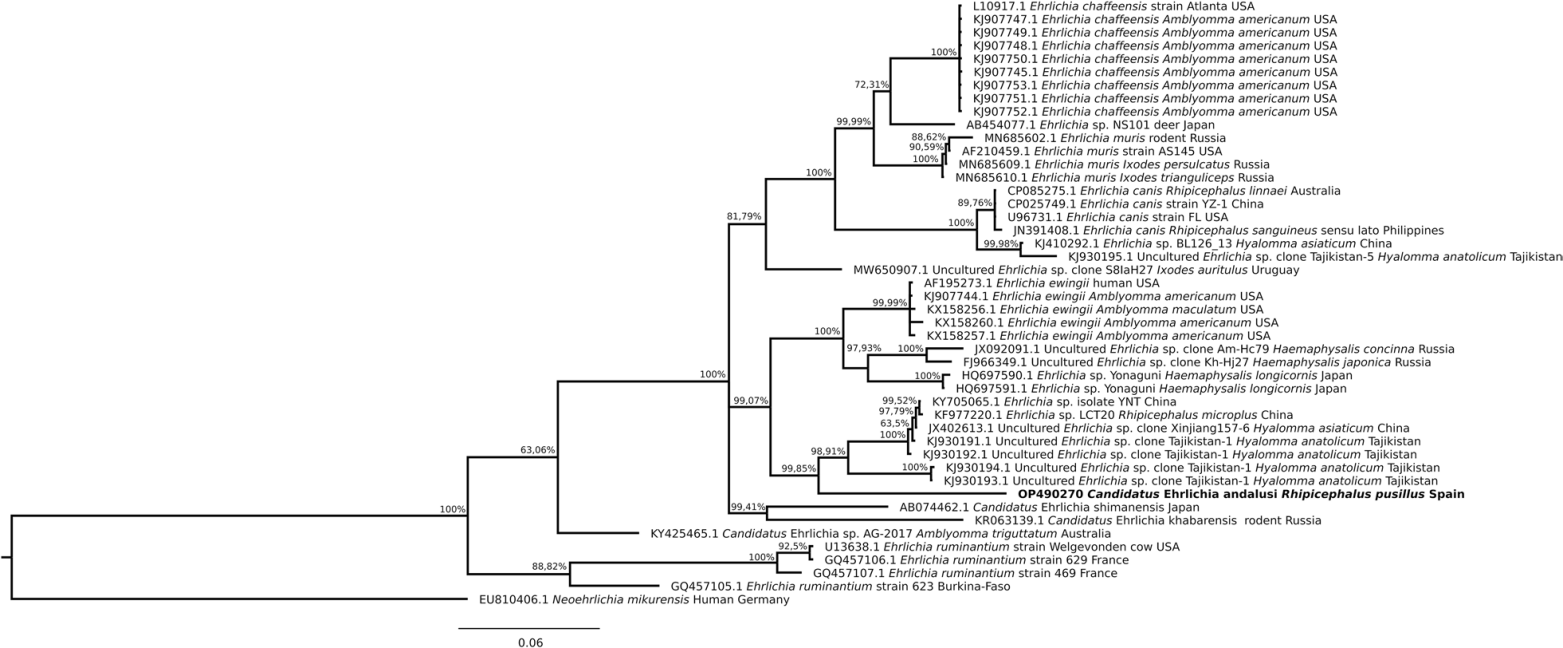
Phylogenetic tree clustering of the partial *groEL* of *Ehrlichia* spp. The tree was obtained using a Hasegawa–Kishino–Yano substitution model with gamma-distributed rate variation across sites (HKY + G) with the software MrBayes 3.2.7 [14] by Bayesian approach with Markov Chain Monte Carlo sampling (10,000,000 generations sampling every 1000 steps). This analysis involved 47 nucleotide sequences. The nucleotide sequence of *Neoehrlichia mikurensis* was used as an outgroup. The isolate identified in this study is indicated in bold

**Fig. 3:**
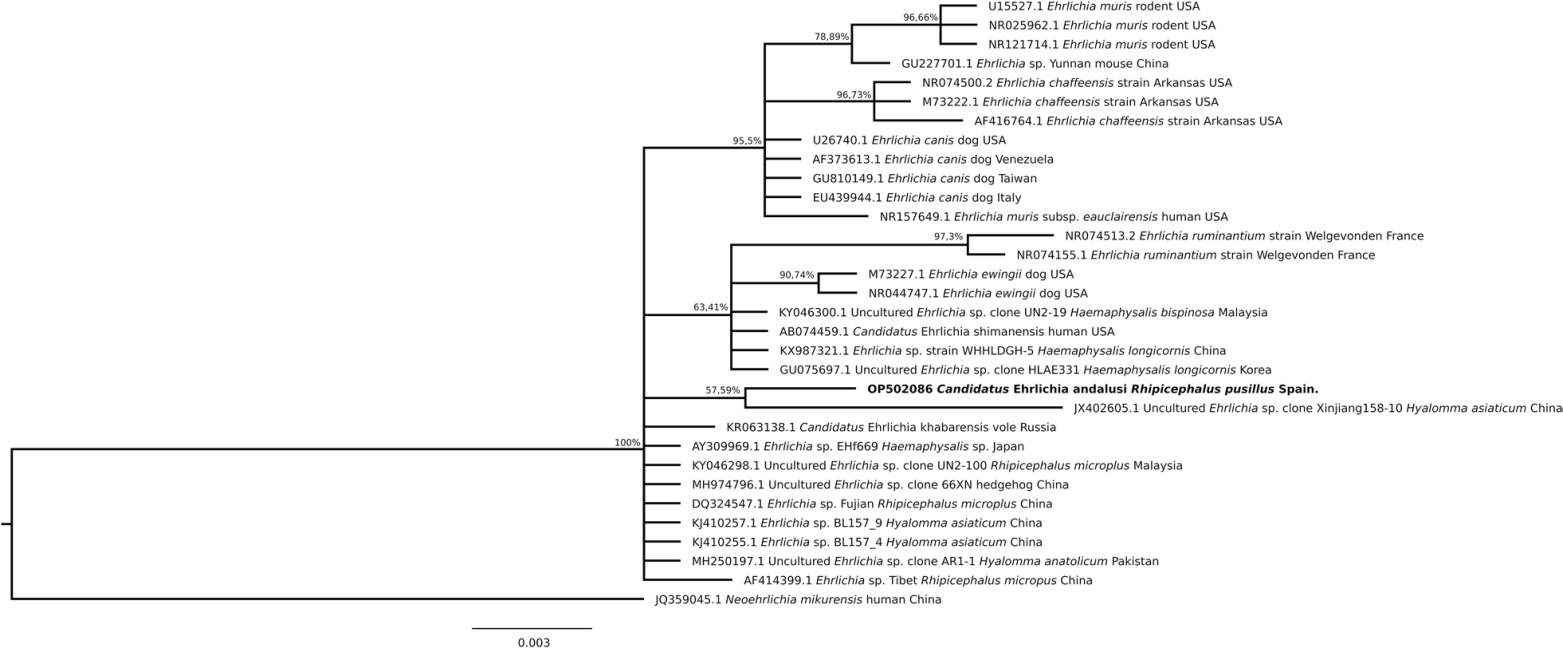
Phylogenetic tree clustering of the partial *16S* RNA of *Ehrlichia* spp. The tree was obtained using HKY + G with the software MrBayes 3.2.7 [14] by Bayesian approach with Markov Chain Monte Carlo sampling (10,000,000 generations sampling every 1000 steps). This analysis involved 66 nucleotide sequences. The nucleotide sequence of *Neoehrlichia mikurensis* was used as an outgroup. The isolate identified in this study is indicated in bold

Phylogenetic trees constructed with partial sequences of the *groEL* and 16S rRNA genes had similar topologies (Figs. 2, 3). The *groEL* sequences formed a clade with sequence KJ930194 detected in *H. anatolicum* from Tajikistan [25], which was clearly separate from the main *Ehrlichia* species. Similarly, the 16S rRNA sequences of *Ehrlichia* sp. obtained in this study formed a clade with sequence JX402605 obtained from *Hyalomma asiaticum* from China [28]. The genetic distances and phylogenetic relationships indicated that a novel *Ehrlichia* species had been found, which is named herein as ‘*Candidatus* Ehrlichia andalusi’. Interestingly, the positive pools comprised ticks collected from wild rabbits from different hunting areas in eastern and western Andalusia (Fig. 1). Since wild rabbits are territorial and live close to their warrens, and their home range is not larger than 10 ha [36], the detection of this novel species in ticks from three geographically separated wild rabbit populations suggests that it may be distributed throughout southern Spain. In support of this hypothesis, no translocations of wild rabbits have been carried out in these hunting areas according to data collected by the gamekeepers.

Novel *Ehrlichia* species and strains have been reported worldwide during the last decades, suggesting that there are several knowledge gaps in the epidemiology and phylogeny of these zoonotic bacteria, especially regarding their sylvatic cycles. Most of these novel organisms were reported for ticks that feed on both domestic and wild animals in South American [37–43] and Asian countries [28, 30, 32, 44]. However, reports of novel *Ehrlichia* species are very scare for Europe, and mainly restricted to ticks collected from wild animals. *Ehrlichia* sp. HF strain was detected in *Ixodes ricinus* collected from the European wood mouse (*Apodemus sylvaticus*) in France [45], as well as in *Ixodes apronophorus*, *Ixodes ricinus* and *R. sanguineus* collected from dogs and foxes in Romania [46, 47]. In addition, a strain similar to *Ehrlichia chaffeensis* and *Ehrlichia muris* was detected in song thrush (*Turdus philomelos*) from Hungary [48].

Since all developmental stages of *R. pusillus* are known to feed on lagomorphs, especially wild rabbits [49, 50], this tick may have a restricted host range. However, it has been sporadically reported in other mammals, such as rodents, ungulates, carnivores and humans [49]. Considering that no transovarial transmission of *Ehrlichia* spp. has been reported in ticks [7], the detection of ‘*Candidatus* E. andalusi’ in *R. pusillus* that were feeding on rabbits may be an accidental finding that is not related to lagomorph populations. Unfortunately, as we were unable to obtain tissue or blood samples from the hunted wild rabbits and hares, we were unable to further examine the role of these lagomorph species in the epidemiology of this pathogen. In this regard, future studies are warranted to investigate the presence of this pathogen in populations of hosts of *R. pusillus*.

Although several *Ehrlichia* species are considered to be pathogenic for humans and animals [6, 51], information on their presence in host or vector populations in Europe is limited. Our results suggest that *Ehrlichia* species of unknown pathogenicity are circulating in wild animal populations or in the ticks that they harbour, which may be of concern for human and animal health. Further studies are needed to determine the presence, prevalence and reservoir range of the *Ehrlichia* species present in Mediterranean ecosystems, and to unravel their epidemiology, pathogenicity and phylogenetic relationships.

## Data Availability

The data that support the findings of this study are available from the authors upon reasonable request.
